# Advances and Prospects in Vaccine Development against Enterococci

**DOI:** 10.3390/cells9112397

**Published:** 2020-11-02

**Authors:** Ermioni Kalfopoulou, Johannes Huebner

**Affiliations:** 1 Institute for Medical Microbiology, Immunology and Hygiene, Technical University of Munich, 81675 Munich, Germany; ermioni.kalfopoulou@tum.de; 2 Division of Paediatric Infectious Diseases, Dr. von Hauner Children’s Hospital, Ludwig Maximilians University, 80337 Munich, Germany

**Keywords:** enterococci, *Enterococcus faecalis*, *Enterococcus faecium*, VRE, vancomycin-resistant enterococci, vaccine, polysaccharide, glycoconjugate vaccine, antibodies

## Abstract

Enterococci are the second most common Gram-positive pathogen responsible for nosocomial infections. Due to the limited number of new antibiotics that reach the medical practice and the resistance of enterococci to the current antibiotic options, passive and active immunotherapies have emerged as a potential prevention and/or treatment strategy against this opportunistic pathogen. In this review, we explore the pathogenicity of these bacteria and their interaction with the host immune response. We provide an overview of the capsular polysaccharides and surface-associated proteins that have been described as potential antigens in anti-enterococcal vaccine formulations. In addition, we describe the current status in vaccine development against enterococci and address the importance and the current advances toward the development of well-defined vaccines with broad coverage against enterococci.

## 1. Introduction

Enterococci are Gram-positive, facultative anaerobic oval cocci and lactic acid producers that form chains of various lengths [1]. Even at the early life stages, enterococci colonize the gastrointestinal tract of humans as commensal bacteria without affecting the host [2]. Some enterococcal strains have also been used as probiotic agents due to their alleged beneficial effects in irritable bowel syndrome, antibiotic-induced diarrhea, and other gastrointestinal diseases. In addition, some enterococci have been proposed to exhibit anticarcinogenic, hypocholesterolemic, and immunomodulatory properties [3,4]. However, under some circumstances, the harmonic relationship with the host can be disrupted, provoking a series of serious diseases [5]. The ability of these bacteria to endure extreme pH conditions and a wide range of temperatures and salt concentrations enables them to colonize a variety of niches and persist in hospital settings. Unlike other bacteria, they are highly tolerant of sodium azide and concentrated bile salts [6]. The increased prevalence of enterococcal infections in humans is mainly attributed to their acquired and intrinsic resistance to antibiotics but also to their ability to acquire virulence factors [7]. In addition, the biofilm-forming capacity of enterococci contributes to their persistence during infection and increases their ability to withstand difficult growth conditions [8].

In this context, this review will describe the origins of enterococcal infections and address current difficulties in the treatment of these multiresistant pathogens, which underscores the necessity for the development of alternative therapeutic regimens. Moreover, the pathogenicity of these bacteria and their interaction with the host immune response will be explored, knowledge of which is of critical importance in vaccine development. In addition, an overview of the up-to-date polysaccharide and protein enterococcal antigens described in the literature will be provided. Finally, the prospects and pitfalls in vaccine development against enterococci will be discussed.

## 2. Enterococcal Infections

Although they usually pose no threat to healthy individuals, enterococci are typical opportunistic pathogens associated with hospital-acquired infections, making them a serious threat for immunocompromised patients. In particular, enterococci can cause serious diseases, including endocarditis, bacteremia, and meningitis, as well as intra-abdominal, wound, and urinary tract infections [9]. The first incidence of endocarditis caused by *Enterococcus faecalis* was reported in 1899, and since then, numerous studies have tried to shed light on this pathogen [10]. The two most clinically relevant enterococcal species are *E. faecalis* and *Enterococcus faecium*, with the highest incidences being initially attributable to *E. faecalis* [5]. During the first wave of enterococcal infections in the late 1970s in the United States (US), *E. faecalis* was the leading clinical enterococcal species [1]. However, during the last two decades, *E. faecium* has become the leading species responsible for enterococcal infections both in US and European hospitals, probably due to its high incidence of antibiotic resistance compared to *E. faecalis* [11]. Currently, a new wave, caused by vancomycin-resistant enterococci (VRE), is affecting not only the US but also Europe [1]. Cassini et al. in a recent study estimated 16,146 (95% uncertainty interval, 13,206–19,334) cases of VRE infections in the EU and European Economic Area in 2015 and an incidence of 1081 (891–1292) attributable deaths [12]. Southern European countries have reported the highest rates of VRE associated with nosocomial infections in Europe [13]. In 2017, the World Health Organization published a list of 12 antibiotic-resistant pathogens that pose the greatest threat to human health, with *E. faecium* being classified as a high priority for the development of new treatments [14].

## 3. Translocation and Colonization

Enterococci, as natural colonizers of the gastrointestinal tract, comprise only a small portion of the healthy gut microbiota. They can spread beyond the gastrointestinal niche into the bloodstream, translocate, and attach to other sites, with subsequent initiation of infection [15]. Exposure of hospitalized patients to antibiotics against Gram-negative bacteria distorts the gut microbiota, increasing the prevalence of mostly VRE in the gastrointestinal tract [1]. Under healthy conditions, lipopolysaccharide and flagellin from Gram-negative bacteria induce the production of REGIIIγ. REGIIIγ suppresses the overgrowth of the Gram-positive bacteria, including *E. faecium*. Elimination of the Gram-negative bacteria population using antibiotics decreases REGIIIγ, leading to overgrowth of VRE in the gastrointestinal tract [16,17]. Similar shifts in the gut microbiota have also been reported in patients undergoing allogeneic hematopoietic stem cell transplantation, where the VRE prevalence in the gut was followed by enterococcal bloodstream infections [18].

Upon entering the systemic circulation, enterococci can reach the collagen-rich valvular and aortic tissues. Distortion of the vascular endothelium can expose extracellular matrix material and cause the formation of a sterile thrombotic vegetation, which is prone to bacterial colonization [19]. Catheter placement is an invasive procedure that increases fibrinogen levels in the bladder lumen due to the inflammation caused by the tissue damage. The fibrinogen deposits on the implanted catheter, acting as a nutrient for bacterial growth and promoting biofilm formation [20,21].

## 4. Host Immune Response against Enterococcal Infections

Limited advances have been made in the elucidation of the host immune response against invasive enterococcal infections. The innate immune system constitutes the first line of defense against pathogen invasion. This type of defense depends on the recognition of the pathogen-associated molecular patterns (PAMPs), which are solely present in the pathogens [22]. PAMPs are recognized through pattern recognition receptors, e.g., the components of the complement system and the Toll-like receptors (TLRs) [22]. There is evidence that TLR2 plays an important role in the innate immune response against Gram-positive bacteria by recognizing peptidoglycan and LTA and by interacting with CD14 [23]. Leendertse et al. showed in an *E. faecium* peritonitis mouse model that *E. faecium* is recognized through TLR2, mediating neutrophil influx to the site of infection and bacterial clearance [24]. In the same model, it was also found that peritoneal macrophages [25], neutrophils [24], and the complement system [26] are essential for the rapid eradication of this bacterium in the early stages of the infection.

Apart from this direct interaction of the pathogen with the phagocyte, there is also an indirect pathway mediated through a class of molecules called opsonins, comprised of immunoglobulins and complement components [27]. Activation of the alternative complement pathway elicits deposition of the complement component C3b on the bacterial surface, which is subsequently recognized by complement receptors on the phagocytes [28]. On the other hand, IgGs trigger the FcγRs and activate the classical complement pathway, resulting in the uptake of the bacteria by the neutrophils [28]. In encapsulated Gram-positive bacteria, like enterococci, the combination of these two mechanisms is crucial for efficient phagocytosis of the bacteria [28,29,30]. A protective immune response against enterococci requires both antibodies and complement for the successful phagocytosis through polymorphonuclear neutrophils (PMNs). For this purpose, the opsonophagocytic assay, by combining these three components, is a reliable surrogate of the protective immune response in order to address the efficacy of enterococcal vaccines [31,32,33]. Immediately upon formation of the phagosome, its maturation starts, and the phagosome subsequently is fused with the lysosome for the formation of a microbicidal organelle, named phagolysosome [27]. In a study, Arduino et al. observed a difference in susceptibility of different enterococcal species to phagocytosis by PMNs. In particular, it was found that 13 out of the 26 *E. faecium* strains tested were resistant to phagocytosis, which was related to a decreased internalization by PMNs. This event may be attributable to a carbohydrate structure that is not sialic acid but that was not isolated or characterized in this study [34]. There have also been reported cases where phagocytic cells failed to kill enterococci, an event that could transform them into vehicles for the translocation of enterococci across the intestinal wall and their dissemination into distant organs [35,36,37]. The incompetence of the immune system to kill the intracellular enterococci may lead to their systemic spread [9].

## 5. Antibiotic Resistance and Options for Treatment

Enterococci possess intrinsic resistance to several antibiotics and may develop acquired resistance through sporadic mutations or by the acquisition of exogenous resistance genes (i.e., by pheromone-sensitive plasmids, broad host range plasmids, or through transposon movement) [7]. Resistance transfer from enterococci to other Gram-positive bacteria, like staphylococci, has also been reported in vitro, and indications for an in vivo exchange exist as well [9,38]. The prevalence of virulence and resistance genes in enterococci of the oral cavity enables the spread of these traits to other species in this environment [39].

The majority of the *E. faecium* clinical isolates are ampicillin-resistant [1]. In most cases, resistance to β-lactam antibiotics in enterococci is attributed to the expression of low-affinity penicillin-binding proteins (PBPs) [40]. In vitro data propose that co-administration of penicillin with aminoglycosides has a synergistic effect by inhibiting the synthesis of the cell wall, thus promoting the uptake of the aminoglycoside [1,41]. Even so, there are instances where this scheme is inadequate, especially when high-level resistance to aminoglycosides is encountered [42].

In the past, the glycopeptide vancomycin was mainly used for the treatment of infections caused by β-lactam or aminoglycoside-resistant enterococci. In 1986, the first vancomycin-resistant enterococcal strain was isolated [43]. Modifications of the d-alanyl-d-alanine terminus of the peptidoglycan precursors to d-Ala-d-lactate or d-Ala-d-serine reduce the affinity of the glycopeptides to the peptidoglycan precursors in VRE [44]. The van gene clusters are responsible for this type of resistance, with VanA and VanB being the most prevalent in Europe, providing a series of enzymes that facilitate the signal transduction, the synthesis of the modified molecules, and their subsequent ligation to the precursors, as well the silence of the alternative biosynthetic pathway [13,44]. Each of these gene clusters is related to different antibiotic susceptibility levels. In particular, the VanA cluster provides high-level resistance to vancomycin and teicoplanin, whereas strains possessing the VanB cluster retain their susceptibility to teicoplanin [45]. In a recent study, 200 enterococcal isolates of human and animal origin were analyzed, revealing the prevalence of teicoplanin- and vancomycin-resistant strains in domestic animals, together with the co-existence of virulence traits [46].

Quinupristin/dalfopristin, daptomycin, tigecycline, and linezolid have entered the clinical practice as alternative antibiotic agents to fight VRE infections. However, resistance to these agents has already been reported [13]. Resistance to quinupristin/dalfopristin includes enzymatic acetylation of the drugs [47,48], methylation of the 23S rRNA [49], and efflux pumps [49,50]. Clinical data suggest that quinupristin/dalfopristin could be beneficial in the treatment of *E. faecium* endocarditis but have an unfavorable toxicity and administration profile [51,52]. The two cell membrane proteins GdpD (glycerophosphoryl diester phosphodiesterase) and LiaF (lipid II cycle-interfering antibiotics protein) are associated with resistance to daptomycin [53]. Daptomycin is approved for skin and soft-tissue VRE infections, and its efficacy against infective endocarditis, either as a monotherapy or in combination with aminoglycosides, ampicillin, or tigecycline, should be further investigated [54,55]. Resistance to linezolid has been attributed to mutations in the domain V or alterations in the methylation of the 23S rRNA [56,57]. Generally, linezolid is recommended as an alternative treatment for endocarditis caused by VRE when other therapeutic options are not available, mainly due to its bone-marrow toxicity [54,55]. On the whole, the therapeutic options to encounter these challenging bacterial infections rely on synergistic agents and are limited due to the presence of multi-resistances and toxic side effects.

## 6. Serotyping of Enterococci

In 1933, Rebecca Lancefield described the serogroups for streptococci, classifying enterococci as group D streptococci [58]. The initial attempt to establish a system to serotype enterococci according to their cell wall type antigens was conducted by Sharpe M.E. in 1964 [59]. In 1992, Maekawa et al. used sera raised against serovars in order to analyze a collection of 832 *E. faecalis* strains. From this collection of bacteria, only 77% were typable into 21 distinct serovars of *E. faecalis* [59]. This classification system was based on sera obtained upon immunization of rabbits with formalin-killed bacteria, thus providing no information regarding the defined antigenic content of these bacteria (e.g., capsules or other cell-wall related antigens) [59].

In 2004, Hufnagel et al. were able to classify 66% of a collection of 29 *E. faecalis* clinical isolates into four capsular polysaccharide serotypes, named CPS-A, -B, -C, and -D. The classification was performed by immunological selection, using sera raised against the capsular polysaccharides of four representative strains and genetic methods [60]. In a further expansion of this study, serotyping of 157 clinical and laboratory *E. faecalis* isolates from four different countries was performed, where the authors were able to categorize only 42% of the isolates into one of the four serotypes [61]. Hancock and Gilmore identified a cps locus of 11 open reading frames that were responsible for the synthesis of a capsular polysaccharide from *E. faecalis* Type 2 [62]. All the serotypes, CPS-A to -D, possess the open reading frames cpsA and cpsB, indicating the importance of these two genes for *E. faecalis* [60]. On the other side, only the CPS-C and -D serotypes possess the cpsC to cpsK, with the cpsF being present only in some CPS-C strains, resulting in the different antigenicity of the two serotypes [60,63]. Since seven of the nine genes of the cps locus are important for the production of the capsular polysaccharide, the CPS-A and -B serotypes do not express this polysaccharide [64]. Theilacker et al. identified the polysaccharide produced by the cps locus, named diheteroglycan (DHG) [65]. A few years later, the study by McBride and co-workers demonstrated that half of the CPS-C strains examined were more virulent compared to the CPS-A and -B strains. This feature is attributable to the presence of multiple virulence and antibiotic-resistant traits in CPS-C, as well as to capsular polysaccharides that play a critical role in the host–pathogen interaction [66].

Overall, to date, a limited number of studies have addressed the serotyping of enterococci. The establishment of an enterococcal serotyping system would determine the coverage and the clinical relevance of the putative immunogens.

## 7. Enterococcal Polysaccharides and Proteins as Potential Vaccine Candidates

A current challenge in the treatment of enterococcal infections in the clinical setting is their resistances to most conventional antibiotics [67]. This underscores the necessity for the development of new types of treatment or prevention, such as passive and active immunotherapies. As mentioned above, capsular polysaccharides, cell wall polysaccharides, and cell-surface associated protein antigens can serve as targets for the development of immunotherapies.

The cell wall of Gram-positive bacteria is primarily comprised of a peptidoglycan layer, consisting of branches of *N*-acetylmuramic acid-(β1-4)-*N*-acetylglucosamine repeating units, which are cross-linked through short peptide bridges [68]. In enterococci the peptidoglycan layer is decorated with a variety of molecules, which are either covalently bound to the peptidoglycan layer (i.e., polysaccharides, teichoic acids, and surface-anchored proteins) or anchored to the plasma membrane (i.e., lipoteichoic acids and lipoproteins) [69].

### 7.1. Enterococcal Polysaccharides

**Figure 1:**
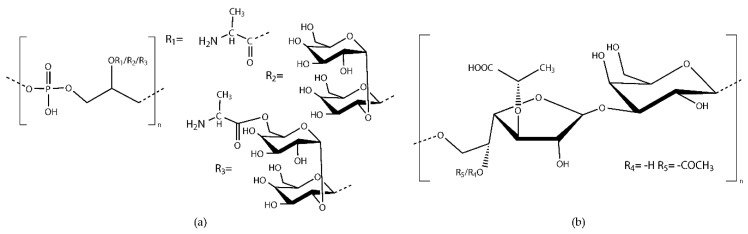
Chemical structure of (**a**) the 1, 3-polyglycerolphosphate backbone of LTA isolated from *E. faecalis* 12030, substituted at the position C-2, with R1 = d-Alanine, R2 = kojibiose, or R3 = alanylated kojibiose [73], and (**b**) DHG isolated from *E. faecalis* Type 2, unsubstituted (R4) or acetylated (R5) [83].

Antibodies targeting capsular carbohydrates have been shown in several studies to promote PMN-mediated killing of *E. faecalis* and *E. faecium* and to protect mice against enterococcal infections [60,70,71]. In 1999, Wang et al. identified a novel polysaccharide that was present in *E. faecalis* and a vancomycin-resistant *E. faecium* strain [72]. Antisera raised to this polysaccharide were able to mediate opsonic killing in vitro and protect against *E. faecalis* and *E. faecium* bacteremia [31,71]. The structural characterization of this polysaccharide was performed by Theilacker et al., revealing a lipoteichoic acid (LTA) consisting of glycerolphosphate repeating units substituted at position C-2 with d-alanine, kojibiose, or d-alanylated kojibiose residues (Figure 1a) [73]. While LTA has a glycolipid anchor in the membrane, wall teichoic acids have a backbone of glycerolphosphate or ribitolphosphate repeating units covalently attached to the peptidoglycan layer by a phosphodiester bond [74]. The same polyglycerolphosphate backbone of LTA is also present in many other clinically important Gram-positive pathogens, such as staphylococci and some streptococci. Theilacker et al. proved that antibodies targeting this preserved backbone are opsonic and protective against *E. faecalis* and *Staphylococcus epidermidis* bacteremia and also confer protection against *Staphylococcus aureus* infection [75]. Another polysaccharide with potential immunogenic properties anchored to the peptidoglycan layer is the enterococcal polysaccharide antigen (Epa) [76]. The Epa is synthesized by the epa locus and contains a rhamnan backbone decorated with phosphopolysaccharide chains of teichoic acids [77,78]. This polysaccharide has been suggested to play a role in biofilm formation, resistance to neutrophil-mediated phagocytosis, virulence in a mouse peritonitis model, and phage infection [77,79,80,81].

Although LTA is present in all enterococcal serotypes, it is only surface-exposed in the CPS-A and CPS-B serotypes, resulting in the susceptibility of these serotypes to opsonization by the sera raised against LTA [65]. Serotypes CPS-C and CPS-D possess a capsular polysaccharide, which masks LTA, dominating in their surface composition and resulting in a different serological recognition compared to serotypes CPS-A and CPS-B [65]. This immunogenic capsular polysaccharide DHG was initially identified by Pazur et al. [82]. The structural elucidation of DHG was accomplished by Theilacker et al. and Krylov et al., revealing a repeating unit of →6)-β-galactofuranose-(1→3)-β-d-glucopyranose-(1→ with *O*-acetylation in position 5 and lactic acid substitution at position 3 of the Galf residue (Figure 1b) [65,82,83]. In the former study, it was also shown that rabbit serum raised against DHG mediates opsonophagocytic killing of the encapsulated strains in vitro and also reduces the bacterial load in livers and kidneys of mice challenged with *E. faecalis* strains of the CPS-C and CPS-D serotypes [65]. It was also suggested that passive or active immunotherapy targeting DHG could provide protection against enterococcal infections caused by the encapsulated *E. faecalis* strains [65].

Classic vaccine production is based on the isolation of polysaccharides from bacterial cultures, whereas modern vaccinology has also attempted synthetic approaches. The final synthetic products have a high batch-to-batch reproducibility, lack bacterial contaminants, and possess a clearly defined chemical structure, which can be easier characterized, providing, thus, better knowledge of the immune-response–oligosaccharide-structure relationship [84]. The antigenic heterogeneity of LTA necessitates structure-activity studies for the elucidation of the immunogenic epitopes. In this case, synthetic groups of LTA, i.e., the D-alanine kojibiose functionalized LTA from *E. faecalis*, and TA fragments would be useful tools [85,86]. In an attempt to develop a vaccine candidate targeting LTA by synthetic approaches, Laverde et al. identified a synthetic teichoic acid, WH7, able to absorb the opsonic activity of antibodies raised against enterococcal LTA. This synthetic oligomer is a promising vaccine candidate against *E. faecalis* and other Gram-positive bacteria [87,88]. Synthetic octamers of the DHG backbone, which lack the acetyl and lactic acid substituents, that conjugated to a classic carrier protein were also able to elicit opsonic and protective antibodies against encapsulated enterococcal species. In this study, we also proved that the length of the synthetic sugar mimetics and the pattern of the repeating units affect the recognition of the sugar mimetic by the immune system [89].

### 7.2. Enterococcal Proteins

The enrolment of the bacterial cell-wall-related and secreted proteins in the bacterial adherence, internalization, toxicity, adaptation to environmental changes, and evasion of the host defense system contributes to their pivotal roles in host–pathogen interactions [90]. The enterococcal vaccine candidates of protein origin described in the literature are summarized in Table 1.

Three secreted virulence factors have been identified so far in enterococci, named cytolysin (Cyl), gelatinase (GelE), and the secreted antigen A (SagA) [91]. SagA was initially identified in *E. faecium* by Teng et al. and has been shown to be essential for bacterial growth as well as to bind a number of extracellular matrix proteins, including fibrinogen, collagen type I, collagen type IV, fibronectin, and laminin [90]. In a recent study, Paganelli et al. identified SagA as the major secreted protein during biofilm formation and studied its susceptibility to degradation, its localization in the biofilm matrix, and its contribution to biofilm formation of *E. faecium* [92]. Kropec et al. demonstrated that immunization with recombinant SagA induces opsonic antibodies against vancomycin-resistant *E. faecium* strains and promotes bacterial clearance in mice challenged with the same bacterial strains. These results suggest that active immunotherapy using only SagA or SagA conjugated with polysaccharides could serve as a promising vaccine candidate against enterococcal infections [93,94]. Lately, GelE was also introduced as a putative vaccine candidate [95].

Another class of virulence factors is the microbial surface components recognizing adhesive matrix molecules (MSCRAMMs), such as Ace [19] and Acm [96], and the pilus proteins that promote biofilm formation [97,98]. Singh et al. reported that immunotherapies targeting the collagen adhesin Ace exhibit varying effectiveness against infective endocarditis and proposed that a robust protection could probably be succeeded by targeting multiple MSCRAMMs [19]. Moreover, Acm-specific antibodies from the serum of an *E. faecium* endocarditis patient and rabbit antibodies raised against the collagen-binding subsegments of Acm significantly inhibited the adherence of *E. faecium* to collagen in vitro [96,99]. The components of endocarditis and biofilm-associated pilus (Ebp), which initiates the bacterial adhesion to the fibrinogen, have been studied for their immunogenicity. This adhesion promotes biofilm formation, a critical step in the development of endocarditis and Catheter-associated urinary tract infections (CAUTIS) [100]. EbpA, EbpB, EbpC, SrtC, and SrtA have been identified to participate in the formation and assembly of the *E. faecalis* Ebp pilus [100,101]. From these components, only immunization with EbpA, and in particular with its amino-terminal domain (EbpA^NTD^), was able to elicit a protective immunoresponse and prevent mice from the development of *E. faecalis* CAUTIS [21]. This event can be attributable to the critical role of the EbpA-fibrinogen interaction in the initial adherence of the bacteria to the catheter surface [102]. The varying protective efficacy of the different components of the same machinery emphasizes that a proper therapeutic intervention requires a thorough understanding of the underlying molecular mechanisms of host–pathogen interactions made by *E. faecalis* [21].

Although virulence factors are present in many vaccine formulations, any bacterial antigen exposed to the immune system can serve as a potential vaccine candidate [103]. In this context, Romero-Saavedra et al. identified six enterococcal proteins that could serve as potential vaccine candidates against enterococcal infections by the implementation of transcriptomic (AdcAfm and PsaAfm) and proteomic (LysM, DdcP, PpiC, and PBP5) approaches [33,94]. In both studies, rabbits were immunized with the recombinant proteins, and the resulting sera were evaluated in opsonophagocytic assay [33,94]. The sera raised against the proteins were opsonic against the homologous strain (*E. faecium* E155) but also against a collection of *E. faecalis* and *E. faecium* strains [33,94]. Moreover, the sera were found to be protective in a mouse bacteremia model [33,94]. Both results indicate the potential use of these proteins as vaccine candidates with a broad cross-reactivity and serotype-independent coverage against enterococcal infections [33,94].

Two of these proteins, AdcAfm and PsaAfm, are zinc and manganese ABC transporter substrate-binding lipoproteins, respectively. Lipoproteins are substrate-binding proteins that deliver the substrate to the corresponding ABC transporters [104]. The immunogenicity of the ABC transporters has also been studied against *E. faecium* and *Streptococcus pneumoniae* [105,106]. In *S. pneumoniae*, PsaA, a homolog of PsaAfm, was a promising vaccine candidate with broad coverage, administrated either solely or as a carrier protein with a synthetic oligosaccharide from *S. pneumoniae* serotype 14 [105]. On the other hand, a peptidoglycan-binding protein LysM, a d-alanyl-d-alanine carboxypeptidase (DdcP), a peptidyl-prolyl cis-trans isomerase (PpiC), and a low-affinity penicillin-binding protein 5 (PBP5) are surface-exposed proteins, which are associated with peptidoglycan [107]. Although the function of these proteins has not been completely elucidated, these four proteins have been associated with resistance to ampicillin and high salt concentrations, and there are indications for their involvement in bacterial virulence and infection [33,108,109,110,111]. Finally, several of these proteins have been identified in membrane vesicles (MVs) of *E. faecium*, conferring immunogenic properties to the MVs and making them interesting vaccine candidates [107].

## 8. Prospects and Pitfalls in the Development of Immunotherapies against Enterococci

The development of an enterococcal vaccine would benefit patients with increased risk factors, increasing their life expectancy and reducing their length of stay, thereby alleviating the stress on the health care system [112]. For this purpose, studies that establish risk factors in well-defined patient populations are of major importance [113,114]. Although the enterococcal surface proteins are promising vaccine candidates, capsular cell wall components in *E. faecium* can still mask their immunorecognition [115]. All the polysaccharides mentioned above could serve as good antigens in vaccine formulations against enterococcus. However, polysaccharides are poorly immunogenic, triggering T cell-independent immune response, and in most cases are unable to elicit memory B cells [116]. Chemical conjugation of polysaccharides with a carrier protein can overcome these obstacles. In particular, the carrier protein directs the processing of the glycoconjugate by polysaccharide-specific B cells. The processed antigen is presented through the MHC class II molecule to the carrier-peptide-specific T cells, thus provoking T cell-dependent immune responses, affinity maturation, and B cell memory [116]. Currently, several glycoconjugate vaccines against bacterial pathogens have been licensed, confirming their safety and efficacy in the prevention of infectious diseases [117]. Apart from their implementations in the vaccine industry, glycoconjugates have also served as immunogens for the production of polysaccharide-specific monoclonal antibodies (mAbs) in mice [118,119,120].

As discussed above, carrier proteins in glycoconjugate vaccines facilitate the T cell-dependent immune response to the conjugated polysaccharide, which is a T cell-independent antigen. The currently licensed carrier proteins for glycoconjugated vaccines are diphtheria toxoid, tetanus toxoid, CRM197, Haemophilus protein D, and the outer membrane protein complex of serogroup B meningococcus [121]. The discovery and usage of new carrier proteins would endorse the development of multivalent vaccines and benefit vaccine co-administration. In particular, the new carrier proteins could simplify vaccine formulations, broaden the coverage of vaccines, and restore the efficacy of the vaccines that exhibit reduced immunoresponse, i.e., carrier-induced epitopic suppression (CIES) or bystander interferences, due to their co-administration with other vaccines [121,122,123]. In this context, conjugates of polysaccharide and protein virulence factors from the same pathogen, where proteins play a dual role not only as a carrier protein but also as an immunogen, have been proposed [121]. For this purpose, in our recent study, we evaluated the two enterococcal proteins, SagA and PpiC, as antigens and carrier proteins for the enterococcal polysaccharide DHG in order to elicit a cross-species immunoresponse against enterococci. The evaluated glycoconjugates exhibited crossreactivity in ELISA and opsonophagocytic assay against several enterococcal strains, as well as protective efficacy in a mouse sepsis model [124].

Passive immunotherapy using mAbs is an emerging field with many promising candidates to fight these health threats, either by replacing the common antibiotic therapy or by co-administration with antibiotics [125,126]. mAbs are also of great importance for the development of chemically defined vaccines by solving the bottleneck of epitope identification [127]. Despite their short-lasting effect, mAbs possess several advantages over vaccines. In particular, they can have a faster effect, with a lower number of doses, can be produced at the industrial level, and most importantly, can even benefit immunosuppressed individuals [128]. To date, a limited number of mAbs against enterococci exist in the literature. In a recent study from Rossmann et al., two opsonic mAbs targeting enterococci were developed, exhibiting promising results in vivo and in vitro [129]. In this study, it was also proposed that these mAbs are directed against LTA, providing a limited coverage only to the CPS-A and CPS-B *E. faecalis* serotypes, since anti-LTA antibodies fail to opsonize the CPS-C and -D *E. faecalis* serotypes [65,129]. Two other mAbs targeting the enterococcal proteins adhesin to collagen (Ace) and the major component of pili (EbpC) are also in preclinical phase [130,131]. In the former case, the mAb targeting the ligand-binding domain A of Ace inhibited *E. faecalis* adhesion to collagen and conferred protection against endocarditis in passive immunization studies [130]. Although immunization with EbpC did not succeed in providing a protective immunoresponse, the mAb targeting the protein diminished biofilm formation and prevented the establishment of a rat endocarditis infection [21,131]. Interestingly, the radiolabeled mAb against EbpC demonstrated accumulation at the site of infection, enabling molecular imaging [131]. The effectiveness of these mAbs against non-encapsulated *E. faecalis* strains provides further support for the development of mAbs against enterococcal infections.

In addition, further research has to be done on the selection of the immunogenic targets for the development of mAbs since the major barrier of mAb development is the antigenic heterogeneity of clinically relevant pathogens [132]. Studies have pointed out this limitation by challenging the efficacy of passive immunotherapies against several clinical isolates, either by targeting conserved immunogens or several immunogens of different strains of the same species. A passive immunotherapy targeting the aforementioned EbpA^NTD^ was described as being protective against a broad range of *E. faecalis*, *E. faecium*, and VRE clinical strains that express fibrinogen-binding diversity and are related to a plethora of clinical manifestations [102]. In our study, two mouse mAbs were developed targeting the polysaccharide DHG and the protein SagA by immunization with the glycoconjugate DHG-SagA. Both antibodies exhibited high specificity and opsonic killing against several enterococcal strains. Interestingly, the mAb against DHG exhibited lower killing compared to the polyclonal serum raised against the same antigen, revealing a variability in the presence and exposure of the recognizable epitope between the strains [126]. A combination therapy using antibodies that target a variety of antigenic epitopes would provide broad coverage and overcome the lack of diagnostic methods [126]. Similar strategies are being explored against other types of diseases, e.g., cancer, and could also be implemented in infectious diseases [133,134]. A combinational mAb therapy is also supported by the opsonic and protective efficacy of human hyperimmune globulin preparations against multidrug-resistant Gram-positive and -negative bacteria [135].

## 9. Conclusions

The therapeutic options against enterococcal infections are limited due to the increasing number of multiresistant isolates in the clinical setting. For this purpose, vaccines and monoclonal antibodies could bridge this gap and provide variety to the panel of treatment and prevention options. Of great importance is the selection of immunogens that would enable the elimination of the bacteria through opsonophagocytosis but also would define the vaccine coverage. For this purpose, a better understanding of the bacterial pathogenesis and the role of virulence factors can allow new targets to be identified. In addition, a well-defined bacterial serotyping system will determine the importance and clinical relevance of these immunogens. Finally, since this field is currently in development and has not yet reached clinical practice, it could profit from the current advances in glycoconjugation and synthetic vaccines. All the corresponding epitopes could also be utilized as targets for passive immunotherapy agents.

## Figures and Tables

**Table 1:** Overview of enterococcal vaccine candidates of protein origin.

Name	Functional Description	Reference
Ace	collagen adhesin	[19]
Acm	collagen adhesin	[96]
SagA	secreted antigen a, bacterial growth and biofilm formation	[93,94]
AdcA_fm_	zinc ABC transporter substrate-binding lipoprotein	[94]
PsaA_fm_	manganese ABC transporter substrate-binding lipoprotein	[94]
LysM	peptidoglycan-binding protein	[33]
DdcP	D-alanyl-D-alanine carboxypeptidase	[33]
PpiC	peptidyl-prolyl cis-trans isomerase	[33,95]
PBP5	penicillin-binding protein 5	[33]
EbpA	endocarditis- and biofilm-associated pili A	[21]
GelE	gelatinase	[95]
